# Efficacy of Rituximab in a Systemic Lupus Erythematosus Patient Presenting with Diffuse Alveolar Hemorrhage

**DOI:** 10.1155/2017/6031053

**Published:** 2017-11-15

**Authors:** Gabriela Montes-Rivera, Grissel Ríos, Luis M. Vilá

**Affiliations:** Division of Rheumatology, Department of Medicine, University of Puerto Rico Medical Sciences Campus, San Juan, PR, USA

## Abstract

Diffuse alveolar hemorrhage (DAH) is a life-threatening complication of systemic lupus erythematosus (SLE). Although infrequent, its mortality is very high. While there are no established therapeutic guidelines, DAH has been traditionally managed with high-dose intravenous (IV) corticosteroids, cyclophosphamide, and plasma exchange. The efficacy of alternative therapies such as rituximab has been described only in a few cases. Herein, we report a 25-year-old Hispanic man who presented with acute-onset SLE manifested by polyarthralgia, nephritis, seizures, pancytopenia, severe hypocomplementemia, and elevated anti-dsDNA antibodies. His disease course was complicated by DAH. His condition was refractory to high-dose intravenous (IV) methylprednisolone pulses, IV cyclophosphamide, and plasmapheresis. Given the lack of clinical response, he was started on IV rituximab 375 mg/m^2^ weekly for a total of four courses. He rapidly improved after the first two doses. Over the next seven months, he did not present recurrent pulmonary symptoms. Follow-up chest computed tomography did not show residual abnormalities. This case, together with other reports, suggests that rituximab is an effective therapeutic option for DAH in SLE.

## 1. Introduction

Systemic lupus erythematosus (SLE) is an autoimmune systemic illness that may affect several organs, including the lungs. There is a myriad of pulmonary manifestations in SLE that ranges from pleuritis and pneumonitis to catastrophic diffuse alveolar hemorrhage (DAH) [1]. The latter is one of the most feared complications given its potentially high mortality rate [2]. Although DAH has a high morbidity and mortality, optimal management guidelines have yet to be established. Conventional treatment includes intravenous pulse (IV) corticosteroids, cyclophosphamide, and plasmapheresis [3]. The efficacy of alternative therapies for DAH in SLE such as rituximab has been described only in a few cases [4–17]. Herein, we report the case of a 25-year-old man with SLE with DAH who successfully responded to rituximab therapy.

## 2. Case Presentation

A 25-year-old man with no history of systemic illness was hospitalized on August 21, 2016, due to a 1-week history of fever, general malaise, polyarthralgia, abdominal discomfort, and diarrhea. Initial physical examination was unremarkable except for the presence of nasal ulcers and swelling of hands, ankles, and feet. Laboratory findings showed leukopenia (white blood cell count = 2.9 K/uL), lymphopenia (lymphocyte count = 0.60 K/uL), anemia (hemoglobin = 11.5 g/dL), and thrombocytopenia (platelet count = 133 K/uL). Reticulocyte count was elevated at 7.7%, but haptoglobulin levels were normal and Coomb's test was negative. Serum chemistries revealed elevated creatinine levels (1.49 mg/dL) and hypoalbuminemia (2.4 g/dL). Urine analysis revealed proteinuria and hematuria (8–30 red blood cells/high power field). A 24-hour urine collection disclosed proteinuria of 2570 mg and decreased creatinine clearance at 61.5 mL/min. Westergren sedimentation rate was elevated at 124 mm/hr. Anti-nuclear antibodies (ANA) were positive (1 : 160, homogenous pattern), and anti-dsDNA antibodies were elevated (>300 IU/mL). He had marked C3 (14 mg/dL) and C4 (<3 mg/dL) hypocomplementemia. Anti-Ro, anti-La, anti-beta-2 glycoprotein 1 (IgA, IgG, and IgM), and anti-cardiolipin (IgA, IgG, and IgM) antibodies were negative. The lupus anticoagulant test was negative. Viral hepatitis panel and HIV test were negative. Bone marrow biopsy, including immunophenotypic analysis, was negative for lymphoma or leukemia.

On August 29, 2016, the patient was treated with methylprednisolone 1 gram intravenous (IV) daily for 3 days, followed by 1 mg/kg/day of prednisone. Also, he was started on mycophenolate mofetil 500 mg orally twice daily and hydroxychloroquine 200 mg orally twice daily. On September 03, 2016, he developed hypertension (168/111 mm Hg) and one episode of tonic-clonic seizures. Brain magnetic resonance imaging revealed changes consistent with posterior reversible encephalopathy syndrome. Brain magnetic resonance angiography was normal.

On September 08, 2016, he had a marked decrease of hemoglobin levels down to 8.7 g/dL. Chest computed tomography (CT) showed alveolar opacification and ground-glass opacities of the right lung becoming near confluent in the central and lower lung. A similar opacification pattern was observed in the left lung. At this time, the patient had no respiratory distress or hemoptysis. Empiric antibiotics (cefepime and vancomycin) were added. Bronchoscopy performed on September 09, 2016, revealed pulmonary hemorrhage with the presence of hemosiderin-laden macrophages. On this day, prednisone was discontinued, and he was started on methylprednisolone 2 mg/kg/daily (methylprednisolone 60 mg IV every 12 hr) and received cyclophosphamide 900 mg IV. Mycophenolate mofetil was discontinued because of severe dyspepsia. Concurrently, plasmapheresis was started and given for five cycles. On September 10, 2016, the patient presented with bibasilar crackles, and hemoglobin levels dropped to 5.1 g/dL. Therefore, methylprednisolone dose was increased to 125 mg IV every 12 hours. He persisted with hemoptysis and severe anemia which required several blood transfusions. After completing five cycles of plasmapheresis, he was treated again with methylprednisolone pulses (500 mg IV every 12 hours for 3 days). Despite these therapeutic interventions, the patient's condition continued to deteriorate, and on September 15, 2016, he required endotracheal intubation and mechanical ventilation. Hemoglobin levels decreased to 4.8 g/dL, and another course of plasmapheresis was given daily for seven days, then every other day for 10 days. In view that he continued with active pulmonary disease, rituximab 375 mg/m^2^ (690 mg) IV weekly for a total of four courses was started on September 16, 2016. Methylprednisolone dose was decreased to 125 mg every 12 hours. On September 20, 2016, *Klebsiella pneumoniae* was detected on sputum culture for which polymyxin B was added. On September 23, 2016, seven days after first dose of rituximab, clinical improvement of hemoptysis was noted evidenced by less blood suctioned from the endotracheal tube and improved mechanical ventilator parameters. On that day, the second dose of rituximab was administered. On September 25, 2016, the patient was successfully extubated, and thereafter his condition continued to improve. Methylprednisolone dose was decreased to 125 mg IV daily, followed by methylprednisolone 80 mg IV daily. On September 29, 2016, only minimal hemoptysis was reported. The third dose of rituximab was given on October 01, 2016. Mycophenolate mofetil 500 mg orally daily was restarted on October 02, 2016, and its dose was increased as tolerated to 2000 mg orally daily. The last dose of rituximab was administered on October 07, 2016. On the next day, IV methylprednisolone was switched to prednisone 60 mg orally daily. Follow-up chest X-ray revealed complete resolution of the bilateral patchy infiltrates. He was discharged home on prednisone 60 mg orally daily, hydroxychloroquine 200 mg orally daily, and mycophenolate mofetil 2000 mg orally daily.

Over the next seven months, prednisone dose was gradually tapered down to 10 mg daily. He was continued on mycophenolate mofetil and hydroxychloroquine. During this follow-up period, he did not present recurrent pulmonary symptoms. Leukopenia, anemia, and thrombocytopenia resolved. Creatinine levels decreased to normal levels (0.99 mg/dL). Proteinuria decreased to less than 1 g daily. Anti-dsDNA antibodies turned negative, and C3 and C4 increased to normal levels (130 mg/dL and 31 mg/dL, resp.). Chest CT done seven months later did not show any abnormalities.

## 3. Discussion

Diffuse alveolar hemorrhage is a rare but catastrophic complication of SLE that warrants rapid diagnosis and aggressive treatment. Bronchoscopy is the diagnostic gold standard for DAH given that imaging findings consisting of bilateral patchy infiltrates may also be found in infectious etiologies and acute respiratory distress syndrome [18]. Despite its high mortality, no management guidelines have been established, and often, patients are refractory to conventional therapy consisting of pulse corticosteroids, cyclophosphamide, and plasma exchange. Consequently, novel treatments such as rituximab have been attempted [4–17].

To the best of our knowledge, there are fifteen SLE cases complicated with DAH who have been treated with rituximab, including ours [4–17]. The demographic features, clinical manifestations, treatment, and outcomes of these patients are depicted in Table 1. Twelve of the described cases were women, and eleven, including our patient, were young with ages between 20 and 30 years. Ethnicity varied among reported cases; two were Hispanics including our patient. As already stated in the literature, most patients who develop DAH had renal involvement. Eleven patients, including our own, had lupus nephritis. Similar to our patient, the most common serological marker abnormalities at the beginning of DAH were elevated double-stranded DNA antibodies and hypocomplementemia.

Most reported SLE patients with DAH who received rituximab were initially treated with IV corticosteroids, and those with longer disease duration were already on maintenance therapy with oral corticosteroids [4–17]. Given that most of them had renal involvement before the onset of DAH, four were already on mycophenolate mofetil, and five had previously received cyclophosphamide. In nine cases, including ours, the patients were facing life-threatening DAH and failed treatment with high-dose corticosteroids and cyclophosphamide. Six of the reported cases received plasma exchange. Thirteen out of fifteen patients successfully responded to rituximab, and in five cases, including our patient, the time of clinical improvement after rituximab was in less than two weeks. Noteworthy, six patients who improved with rituximab did not receive cyclophosphamide and/or pulse methylprednisolone therapy [4, 5, 9, 12, 13, 15].

Clinical outcomes of SLE patients presenting with DAH and treated with rituximab have been reported in case series. For example, Kazzaz et al. showed that three out of twenty-two patients who received rituximab, among other immunosuppressive treatments, survived [19]. On the other hand, in a case series of 140 SLE patients, three out of eight patients who received rituximab died [20]. However, in these case series, details about clinical manifestations, immunosuppressive treatments, and outcomes are not reported. Nonetheless, we have to acknowledge that cases with unfavorable outcomes are usually not reported with the same frequency as those with positive outcomes.

B-cell-depleting therapies are promising options for the management of SLE. Rituximab, an anti-CD20 monoclonal antibody, acts against the autoreactive B cells that regulate T-lymphocyte-dependent immune responses and dendritic cells [21]. Typically, its use in SLE is considered in refractory cases or even life-threatening situations such as severe lupus nephritis and myelitis and as a second- or third-line therapy in refractory cytopenias, vasculitis, and central nervous system involvement [22]. It can be argued that the clinical response observed in our patient was attributable to corticosteroids, plasmapheresis, and cyclophosphamide, and not to rituximab. However, the immunological changes after rituximab treatment occur early after infusion. A study conducted in lupus nephritis patients treated with rituximab showed that peripheral B cells were not detected by flow cytometry as quickly as one week after therapy in eight out of ten patients [23]. B-cell depletion was associated with a reduction in both ANA and dsDNA antibodies. Furthermore, in renal transplant patients, rituximab increases levels of IL-10 and MIP-1β after only 2 hours of drug infusion [24]. IL-10 is a known anti-inflammatory cytokine, and MIP-1β is a chemoattractant for regulatory T cells, thus inducing a downregulation of the immune response.

The potential role of rituximab for pulmonary hemorrhage is further supported by a murine model that showed the pivotal role of B cells in the pathogenesis of DAH by inducing lung inflammation with pristane [25, 26]. This agent is a natural saturated terpenoid alkane that induces a lupus-like autoimmune syndrome. The bronchiolar lavage from pristane-treated mice showed the presence of monocytes and granulocytes as well as an increased number of CD19+ B cells and CD4+ and CD8+ T cells. These mice were more prone to develop alveolar hemorrhage. Conversely, mice that lacked both B and T cells had significantly reduced frequency of DAH.

In summary, this case, together with other reports, suggests that rituximab might be an alternative drug for the treatment of DAH as it targets B-cell-mediated immune responses proven to be involved in the pathogenesis of this complication. Although frequently added to conventional therapies in refractory cases, the encouraging outcomes presented here set forth the possibility of designing controlled studies to examine the efficacy of rituximab in the induction phase.

## Figures and Tables

**Table 1:** Demographic features, clinical manifestations, treatment, and outcome in SLE patients presenting with diffuse alveolar hemorrhage (DAH) treated with rituximab.

Authors/year of publication	Age, sex, ethnicity	Time from diagnosis of SLE to onset of DAH, years	Cumulative major organ SLE involvement	Previous SLE treatment	Positive serologies at DAH	Treatment	Outcome
*Favorable clinical outcome to rituximab therapy*							
Gillis et al. [4]/2007	24-year-old, female, Pacific Islander	1	NR	IV cyclophosphamide Plasma exchange IVIG IV methylprednisolone Azathioprine	NR	Prednisone 60 mg	Survived
						Hydroxychloroquine	
						Dapsone	
						IV rituximab 375 mg/m^2^ on day 1, 7, 14, and 21	
						Maintenance: azathioprine	

Nellessen et al. [5]/2008	29-year-old, female, North African	12	Nephritis (class III)	Prednisone IV cyclophosphamide Mycophenolate mofetil	ANA Anti-dsDNA Low C3 Low C4	IV prednisolone	Survived, no relapse
						IV cyclophosphamide	
						Plasma exchange	
						Prophylactic antibiotics and antifungals	
						Discharged on: prednisolone and cyclosporine	
						Relapse: same regimen as during first presentation combined with IV rituximab 500 mg (375 mg/m^2^) every 2 weeks for 6 weeks	
						Maintenance: cyclosporine, mycophenolate mofetil, and prednisolone	

Pinto et al. [6]/2009	19-year-old, female, Hispanic	4	Nephritis (class IV)	NR	NR	IV hydrocortisone	Survived
						IV methylprednisolone pulse	
						IV cyclophosphamide	
						IV rituximab 375 mg/m^2^	
						Relapse: IV methylprednisolone pulse	
						IV cyclophosphamide	
						IV rituximab 375 mg/m^2^	
						Maintenance: chloroquine and prednisone	

Narshi et al. [7]/2009	52-year-old, female, NR	12	Nephritis (class IV)	IV methylprednisolone IV cyclophosphamide Prednisolone	ANA Anti-dsDNA Low C3 Low C4	Broad-spectrum antibiotics	Survived, no relapse in 16 months
						IV methylprednisolone pulse	
						IV rituximab 1000 mg on day 9 and 25	
						IV cyclophosphamide	
						Maintenance: prednisolone	

Todd and Costenbader [8]/2009	24-year-old, female, Cambodian	5	Nephritis (class IV)	Corticosteroids Hydroxychloroquine Azathioprine IV cyclophosphamide IV rituximab 375 mg/m^2^/week for 4 weeks, 3 courses 6–9 months apart (for nephritis)	ANA Anti-dsDNA Low C3 Low C4 Anti-Ro/La	IV dexamethasone	No relapse, died after 10 months; cause of death unknown
						Empiric antibiotics	
						IV methylprednisole pulse	
						IV cyclophosphamide	
						Plasmapheresis	
						Recombinant activated factor VII	
						IV rituximab 375 mg/m^2^ 2 infusions, 2 weeks apart	

Pottier et al. [9]/2011	18-year-old, male, Caucasian	6	Nephritis	IV corticosteroids IV cyclophosphamide Mycophenolate mofetil	ANA Anti-dsDNA Low C3 Low C4	IV methylprednisolone pulse	Survived, no relapse in 15 months
						Mycophenolate mofetil	
						Plasma exchange	
						Rituximab 715 mg (375 mg/m^2^) at days 6 and 21	
						Maintenance: prednisolone and mycophenolate mofetil	

Martinez-Martinez and Abud-Mendoza [10]/2012	23-year-old, female, NR	4	Nephritis (class IV)	Methotrexate Prednisone	ANA Anti-dsDNA	IV methylprenisolone pulse	Survived, no relapse in 12 months
						IV cyclophosphamide	
						Discharged on: prednisone and mycophenolate mofetil	
						Relapse: IV rituximab 2 doses of 1000 mg 2 weeks apart	
						Maintenance: prednisone and azathioprine	

Gonzalez-Echavarri et al. [11]/2014	27-year-old, female, Caucasian	2	NR	Hydroxychloroquine	ANA Anti-dsDNA Low C3 Low C4	IV methylprednisolone pulse	Survived, no relapse in 12 months
						IV cyclophosphamide	
						IV rituximab 2 doses of 1000 mg 2 weeks apart	
						Hydroxychloroquine	
						Prednisone	
						Maintenance: prednisone, azathioprine, and hydroxychloroquine	

Esper et al. [12]/2014	37-year-old, female, NR	NR	NR	Deflazacort	ANA Low C3 Low C4	IV methylprednisolone pulse	Survived
						Recombinant activated factor VIII	
						IV rituximab 500 mg x 1	

Tse et al. [13]/2015	52-year-old, female, NR	8	Nephritis, S/P renal transplant	Prednisone Hydroxychloroquine Mycophenolate mofetil For renal transplant: IV methylprednisolone Thymoglobulin Tacrolimus	ANA Low C3	Empiric antibiotics	Survived, no relapse in 6 months
						IV methylprednisolone pulse	
						IVIG	
						Plasma exchange	
						Discharged on: mycophenolate and tacrolimus	
						Relapse: IV methylprednisolone pulse	
						Plasma exchange	
						IVIG	
						IV rituximab 550 mg (375 mg/m^2^) weekly x 3, the 4th dose seven days after discharge	

Na et al. [14]/2015	37-year-old, female, NR	NR	NR	NR	ANA Anti-dsDNA Anti-Smith Anti-Ro Anti-La	Empiric antibiotics	Survived, no relapse in 4 years
						IV methylprednisolone pulse	
						IV rituximab 500 mg (375 mg/m^2^) on days 3 and 10	
						Maintenance: prednisolone	

Aakjær et al. [15]/2017	24-year-old, male, Caucasian	15	Nephritis (class IV)	Prednisolone IV cyclophosphamide Azathioprine Mycophenolate mofetil	Low C3	Mycophenolate mofetil	Survived, no relapse in 8 years
						IV rituximab 2 doses of 1000 mg 2 weeks apart	
						Relapse: IV rituximab 1000 mg yearly for one year, then every 6 months	
						Maintenance: prednisolone, mycophenolate mofetil, hydroxychloroquine, and IV rituximab 1000 mg every 4 months	

Current case	25-year-old, male, Hispanic	0	Central nervous system, nephritis	NA	ANA Anti-dsDNA Low C3 Low C4	IV methylprednisolone pulse	Survived, no relapse in 8 months
						IV cyclophosphamide	
						Plasmapheresis	
						IV rituximab 375 mg/m^2^ weekly for 4 weeks	
						Maintenance: prednisone and mycophenolate mofetil	

*Unfavorable clinical outcome to rituximab therapy*							
Verzegnassi et al. [16]/2010	13-year-old, female, NR	0.3	Nephritis	NA	ANA Anti-dsDNA Low C3 Low C4	Empiric antibiotics	DAH relapse which responded favorably to plasmapheresis
						IV methylprednisolone pulse	
						IV cyclophosphamide	
						IV rituximab 375 mg/m^2^ weekly x 3	
						Maintenance: prednisone	

Ta et al. [17]/2016	16-year-old, female, African American	0.2	Myocarditis, nephritis	NA	ANA Anti-dsDNA Low C3 Low C4	Corticosteroids	Died 25 days after admission
						Plasmapheresis	
						IV cyclophosphamide	
						IVIG	
						IV rituximab (dose not specified)	

SLE: systemic lupus erythematosus; IV: intravenous; ANA: anti-nuclear antibodies; NR: not reported; IVIG: intravenous immunoglobulin; NA: not applicable; ENA: extractable nuclear antigen.
